# Perceptions Regarding COVID-19 Vaccination Among a Representative Pakistani Population Coming to Tertiary Care Cardiac Hospital

**DOI:** 10.7759/cureus.18654

**Published:** 2021-10-11

**Authors:** Saba Hussain, Farheen Ali, Nawal Salahuddin, Musa Karim, Shakeela Naz, Rizwan A Khawaja, Sadaf Rifaz

**Affiliations:** 1 Non-invasive Imaging, National Institute of Cardiovascular Diseases, Karachi, PAK; 2 Infectious Disease, National Institute of Cardiovascular Diseases, Karachi, PAK; 3 Adult Critical Care Medicine, National Institute of Cardiovascular Diseases, Karachi, PAK; 4 Statistics, National Institute of Cardiovascular Diseases, Karachi, PAK; 5 Cardiology, National Institute of Cardiovascular Diseases, Karachi, PAK

**Keywords:** vaccine, pakistan, hesitancy, covid-19, acceptance

## Abstract

Background: The deleterious effects caused by coronavirus disease 2019 (COVID-19) infection have been compounded by COVID-19 vaccine hesitancy throughout the world, including Pakistan. We are lacking representative national data regarding the COVID-19 vaccine acceptance. This study aims to determine COVID-19 vaccination acceptance rates and predictors of its acceptance and hesitancy among a representative Pakistani population.

Methods: This cross-sectional study was conducted at the National Institute of Cardiovascular Disease, Karachi, from March 2021 to April 2021. Participants included patients, and their attendants visiting the outpatient clinics and healthcare workers of the institute. Participants were labeled as ‘acceptant’ or ‘hesitant’ based on their responses of ‘yes’ or ‘no’ and ‘not sure’ on the willingness to get vaccinated, respectively. The Chi-square test was used to calculate the significant association between different variables. A p-value ≤0.05 was set as a level of significance for all statistical analyses.

Results: Overall, 1500 participants were enrolled with a vaccine acceptance rate of only 49%. Factors like male gender, unmarried and employed status, higher education, high socioeconomic class, Punjabi and Sindhi ethnicity, medical professional, and self or family exposure of COVID-19 were positively related to COVID-19 vaccine acceptance. The commonest stated reason for the vaccine hesitancy was distrust in vaccine efficacy or fear of vaccine adverse effects.

Conclusion: Vaccine hesitancy remains a serious challenge in our population, related to multiple demographic and thought factors. Focused actions and modification of these factors are the keys to conclude this COVID pandemic.

## Introduction

After the identification of two cases in China in December 2019, novel coronavirus disease 2019 (COVID-19) has grown into a sweeping pandemic all over the world causing significant negative psychological, socioeconomic, and life-threatening impacts [1,2]. The first case of COVID-19 was confirmed on 26 February 2020 in Pakistan. Afterward, the disease has spread nationwide with currently approximately 829933 confirmed cases and 18070 reported deaths [3]. As the COVID-19 has grown wild, multiple conspiracy theories have also been circulated throughout the time [4,5]. Those confederacies have stormed further after the approval of COVID-19 vaccination causing a disastrous infodemic [6,7]. In the absence of effective therapy for COVID-19, vaccination is one of the most important measures for the prevention of the COVID-19 pandemic and its detrimental effects.

In the literature, surveys of the acceptance and hesitancy regarding COVID-19 vaccination have shown 55 to 90% acceptance rates with relatively higher acceptance in Southeast Asia [8-10]. A study from Pakistan had shown a 66% acceptance rate of COVID-19 vaccine if made freely available [11]. However, many of such studies are online or telephonic surveys that have the major limitation of comparatively literate people using the internet to respond. This bias is also visible with the very small percentage of vaccinated people after its free availability in Pakistan [3,12].

This study was aimed to ascertain the acceptance rate and perspective of COVID-19 vaccination among a representative population coming to our tertiary care center, which has patients coming from all over Pakistan. This study is an attempt to identify the potential barriers to COVID-19 vaccination so that they can be targeted to improve COVID-19 vaccine acceptance and thus to limit the spread of COVID-19 and its devastating impacts.

## Materials and methods

We have conducted this cross-sectional survey from March 2021 to April 2021 at the National Institute of Cardiovascular disease. Our study was approved by the institutional Ethical Review Committee (Reference no: ERC-43/2021). The minimum required sample size was 381 participants, based on the acceptance rate of 55 to 90% of COVID-19 vaccines among the general population, taking the smaller statistics values, at 95% confidence level, and 5% margin of error [9]. Nonprobability consecutive sampling was used. To improve the reliability of the study we have recruited 1500 participants (including visiting outpatients, their attendants, and hospital employees) of either gender of age >18. Persons who were unable to respond because of mental or physical illness and persons in the emergency department or admitted in wards were excluded to prevent stress bias. Verbal informed consent was taken from each participant before enrolment. The data collection was done through a questionnaire defined on the basis of previous studies. The questionnaire was pretested in a small group of participants and then finalized and administered through proforma (Appendix 1). The questionnaire mainly consisted of three components: a) demographic details; b) intention to get vaccinated if the vaccine is freely available and with no proven contraindication; c) reason for the acceptance or hesitancy to the vaccine. The people who agreed upon vaccination were labeled as the ‘acceptant’, while those who were not sure or refused vaccination were labeled as ‘hesitant’. Translators were used in cases where the language was a barrier. The data feeding and analysis were done on SPSS (Statistical Package for the Social sciences) software version 17.0 (SPSS Inc., Chicago, USA). Clinical characteristics were summarized in terms of frequency and percentages for qualitative variables and mean with a standard deviation of quantitative/continuous variables. The Chi-square test was used for statistical comparison of qualitative variables with vaccination acceptance. Only a p-value ≤0.05 was considered significant in all statistical analyses.

## Results

**Figure 1:**
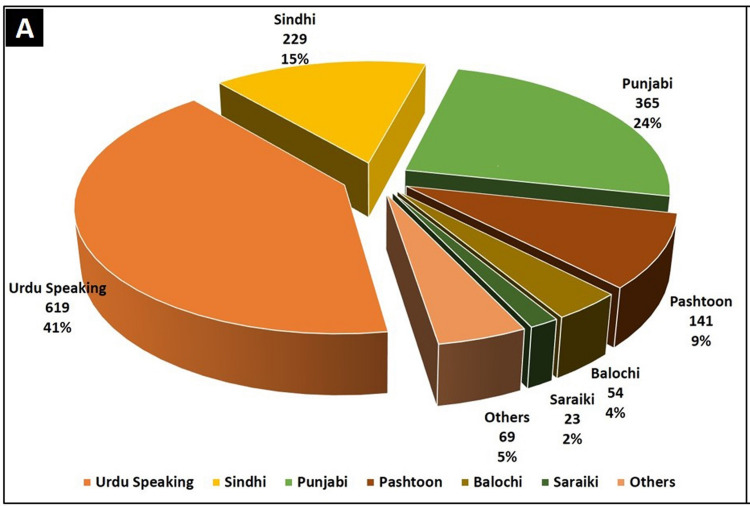
Graphical representation of the ethnic distribution of our study participants. Data source: World Atlas available from https://www.worldatlas.com/articles/ethnic-groups-in-pakistan.html

**Figure 2:**
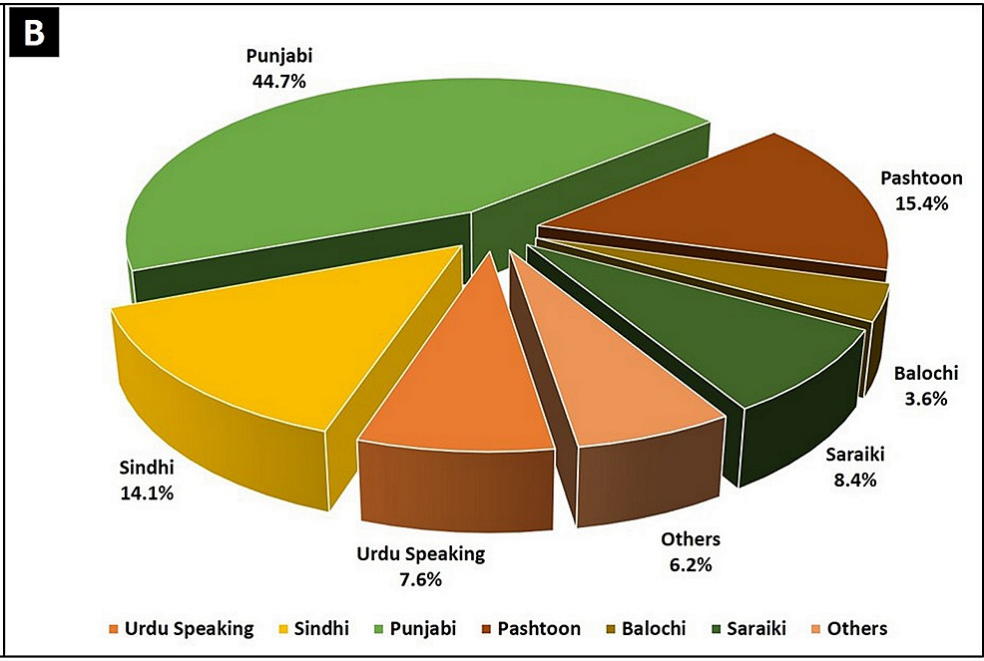
Graphical representation of the ethnic groups of Pakistan. Data source: World Atlas available from https://www.worldatlas.com/articles/ethnic-groups-in-pakistan.html

The median age of our study population was 38 years with a comparable number of female respondents (male to female ratio 1.4:1). Although not the true representative of Pakistan’s ethnic distribution, there was a sizable representation from Pakistan’s major ethnic groups (Figures 1-2).

Overall, 734 (49%) subjects responded ‘Yes’ for the COVID-19 vaccination while 766 (51%) either refused or were not sure. Table 1 shows the correlation of the survey participant’s demographic details and past self or family exposure to COVID-19 infection with the acceptance and hesitancy of the COVID-19 vaccine. Male participants have significantly higher acceptance rates than their female counterparts (53% vs. 43%). Similarly, interviewees that were unmarried, or were having urban belonging or those with high socioeconomic class showed considerably higher acceptance. There was no marked difference among the different age groups (p value= 0.823). Almost 50% of our participants have not studied above 10th grade. Those who have higher education showed significantly higher acceptance (p value= <0.001). A population of 20% of the study group was related to a medical profession in different ways and was found to be substantially more acceptant (p value= <0.001). Subgroup analysis reported those who were employed (medical and non-medical) were markedly more agreed for the vaccination in comparison to the unemployed group (54% vs. 39%). Also, a significant difference was found among the different ethnic groups as people with Punjabi and Sindhi ethnicity were more likely to get vaccinated. Persons who had previously received vaccines for any other infectious disease in their adulthood were way more agreed for the COVID-19 vaccination (p value= <0.001). Finally, acceptance was markedly higher in participants who had prior exposure to COVID-19 infection either self or in the family.

**Table 1:** Correlation of participant’s demographic details with the COVID-19 vaccine acceptance and hesitancy. * - significant p-value (≤0.05 ); COVID-19 - Coronavirus disease 2019; PKR - Pakistani Rupee; Matric - 10^th ^Grade

Characteristics	Total	Agreed for free COVID-19 vaccine			p-value
		Acceptant	Hesitant		
		Yes	No	Not Sure	
Total (N)	1500	734 (48.9%)	542 (36.1%)	224 (14.9%)	-
Age (years)	38.45 ± 12.57	38.5 ± 12.65	38.58 ± 12.7	37.98 ± 12.01	0.824
Young (≤ 40 years)	64.6% (969)	49.3% (478)	35.2% (341)	15.5% (150)	0.823
Middle age (41 to 65 years)	33.1% (496)	48% (238)	38.1% (189)	13.9% (69)	
Elderly (>65 years)	2.3% (35)	51.4% (18)	34.3% (12)	14.3% (5)	
Gender					
Male	57.7% (865)	53.1% (459)	32.5% (281)	14.5% (125)	0.001*
Female	42.3% (635)	43.3% (275)	41.1% (261)	15.6% (99)	
Marital status					
Unmarried	19.1% (287)	55.1% (158)	29.6% (85)	15.3% (44)	0.031*
Married	80.9% (1213)	47.5% (576)	37.7% (457)	14.8% (180)	
Ethnicity					
Urdu Speaking	41.3% (619)	46.4% (287)	37.3% (231)	16.3% (101)	0.016*
Sindhi	15.3% (229)	54.6% (125)	35.4% (81)	10% (23)	
Punjabi	24.3% (365)	54% (197)	32.1% (117)	14% (51)	
Pashtoon	9.4% (141)	41.1% (58)	39.7% (56)	19.1% (27)	
Balochi	3.6% (54)	35.2% (19)	44.4% (24)	20.4% (11)	
Saraiki	1.5% (23)	47.8% (11)	26.1% (6)	26.1% (6)	
Others	4.6% (69)	53.6% (37)	39.1% (27)	7.2% (5)	
Residence					
Urban	80% (1200)	50.9% (611)	34.2% (410)	14.9% (179)	0.004*
Rural	20% (300)	41% (123)	44% (132)	15% (45)	
Socioeconomic class (PKR/month)					
Low (<25,000)	39.6% (594)	36.4% (216)	47.1% (280)	16.5% (98)	<0.001*
Middle (25,000 - 50,000)	41.9% (628)	53.2% (334)	30.9% (194)	15.9% (100)	
High (> 50,000)	18.5% (278)	66.2% (184)	24.5% (68)	9.4% (26)	
Education					
No Formal education	16.2% (243)	32.1% (78)	51.4% (125)	16.5% (40)	<0.001*
Primary (Class-5)	11.7% (175)	36.6% (64)	43.4% (76)	20% (35)	
Secondary (Class 6 to Matric)	22.2% (333)	42.3% (141)	45.3% (151)	12.3% (41)	
Higher (intermediate to graduation)	49.9% (749)	60.2% (451)	25.4% (190)	14.4% (108)	
Occupation					
Employed (Non-Medical)	50.9% (763)	52.7% (402)	31.8% (243)	15.5% (118)	<0.001*
Employed (Medical)	17.8% (267)	56.2% (150)	33.3% (89)	10.5% (28)	
Unemployed	31.3% (470)	38.7% (182)	44.7% (210)	16.6% (78)	
Working status					
Employed	68.7% (1030)	53.6% (552)	32.2% (332)	14.2% (146)	<0.001*
Unemployed	31.3% (470)	38.7% (182)	44.7% (210)	16.6% (78)	
Previously vaccinated other than COVID-19					
No	75.9% (1138)	44.6% (507)	39.3% (447)	16.2% (184)	<0.001*
Yes	24.1% (362)	62.7% (227)	26.2% (95)	11% (40)	
Had COVID-19					
No	94.1% (1411)	47.1% (664)	37.6% (530)	15.4% (217)	<0.001*
Yes	5.9% (89)	78.7% (70)	13.5% (12)	7.9% (7)	
Family member affected with COVID-19					
No	85.6% (1284)	45.1% (579)	40% (513)	15% (192)	<0.001*
Yes	14.4% (216)	71.8% (155)	13.4% (29)	14.8% (32)	

**Figure 3:**
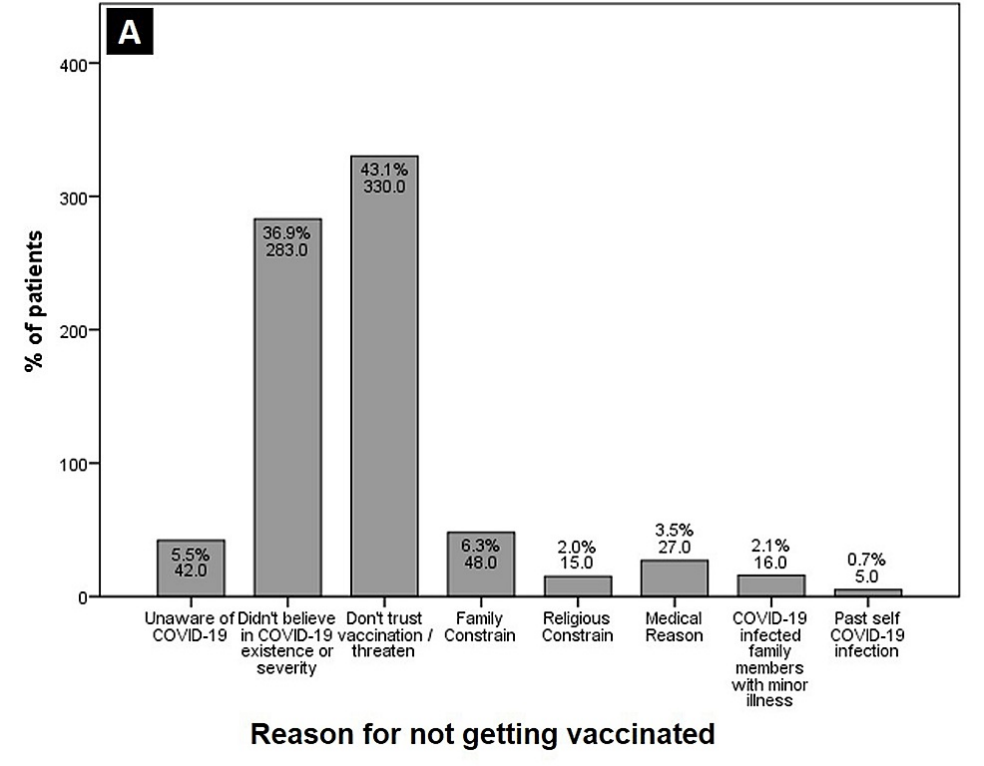
Stated reasons for COVID-19 vaccine hesitancy.

**Figure 4:**
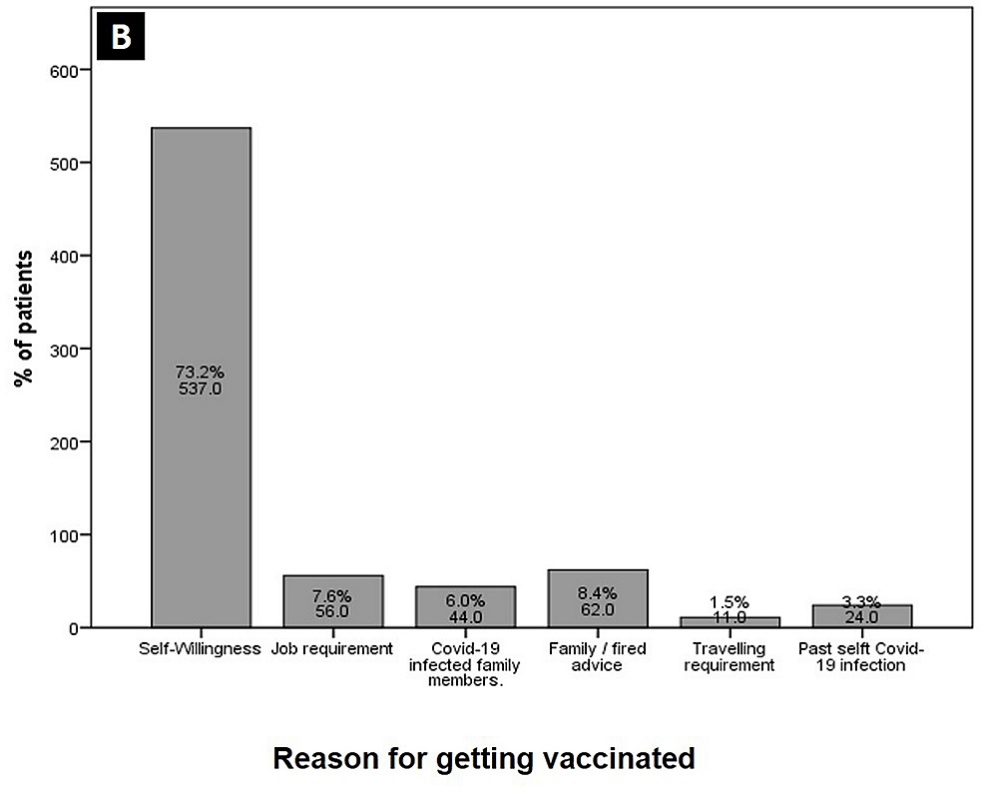
Stated reasons for COVID-19 vaccine acceptance.

When the primary reason was asked for the vaccine hesitancy, most people were either threatened because of wondering rumors against the vaccine or were unsure of the vaccine’s effectiveness. Figures 3-4 show different reasons given for the COVID-19 vaccine hesitancy and acceptance, respectively.

## Discussion

Novel coronavirus 2019 belongs to the Coronaviridae family that is already notorious for causing earlier outbreaks (i.e., SARS-CoV-1 for the 2003 SARS epidemic and MERS-CoV for the 2013 MERS epidemic) [13]. However, despite all the medical and technological advancements, this new coronavirus pandemic has come as the worst nightmare that has led to drastic physical, social, financial as well as psychological upsets all around the world, not only by the disease itself but also by the preventive measures (lockdown, mask use, and social distancing, etc.) that are taken for it [1,14]. All of this has led to the development of vaccines against the COVID-19 virus, which appears to be the best hope at this time to bring this pandemic to an end [15].

Vaccines act by stimulating the body’s immune system against various disease-causing microorganisms [15]. Vaccines against the COVID-19 virus are based upon varied methodological platforms like nucleic acids (DNA and RNA), virus-like particles, peptides, viral vectors, recombinant proteins live attenuated viruses, and inactivated viruses [16]. COVID-19 vaccination is probably the largest and crucial vaccination campaign ever to end up this pandemic. However, this vaccination campaign has been affected negatively throughout the world with a significant hesitancy rate [6,9]. Like other parts of the world, Pakistan has been badly affected by COVID-19, further shattering our weak socio-economic and health structures [2,3]. Unfortunately, as proven by the past experience of the polio vaccine, Pakistan is at high risk of COVID-19 vaccine resistance [7]. There are studies for COVID vaccination hesitancy from around the world and a couple of studies from Pakistan [6-11,17,18]. But most of them are likely biased by the comparatively literate responder to their online surveys, the method used in most such studies. As a significant majority of the Pakistani population have low literacy levels and belong to a lower socio-economic group, in this study we investigated the COVID-19 vaccine acceptance and hesitancy rates along with the factors contributing to its hesitancy in a more diverse and representative Pakistani population. Our study is an attempt to recognize and thus mitigate the barriers to COVID-19 vaccination which may give a greater chance of success to the vaccine initiative.

According to the WHO, vaccine hesitancy is a delay in acceptance or refusal of vaccination despite the availability of vaccination services [19]. In our study, we found almost 51% of people were hesitant about vaccination with an acceptance rate of only 49%. This is contrary to the 66% acceptance found in scarce data from Pakistan [11]. As mentioned before the difference was expected because of more literate responders in those studies. Our acceptance rate is even lower than our neighboring countries and unfortunately is far lower than what is required for herd immunity [6,8,10,20].

Our respondents were from a wide range of ages and we found no significant difference in their acceptance rates based on age. Even, no persistent pattern was found between age and COVID-19 vaccine acceptance in data from around the world [8,9,21]. Unlike the western countries where females were more likely agreed to the vaccination, our male respondents were more acceptant [9,22]. This finding was probably secondary to the comparatively knowledgeable males owing to their work nature while most of our females have limited social encounters. Job requisites might also have contributed to this gender difference. We found the people that were unmarried have a considerably higher intention for vaccination. A possible explanation might be that most of these individuals were much younger so have higher accessibility to the information through the technological devices as well as being less fearful. Similar to other parts of the world, in our study urbanization has been linked with higher acceptance likely secondary to higher literacy and knowledge access [21]. We got interviewees from almost all major Pakistani ethnic groups and have observed that participants that were either Sindhi or Punjabi were remarkably more agreed for the vaccination. No single justification can fit into it and there must be multiple sociocultural factors for this difference. Expectedly, education did have a positive impact with hesitancy rates being higher among our less literate studied samples. This has also been shown by the prior study from Pakistan where higher education levels had higher adherence to preventive measures against COVID [5]. Even global data favors higher education for vaccination acceptance [9,21-23]. Similarly, as seen around the world and in a study from Pakistan, employed participants and especially those related to medical fields were found to be more welcoming to COVID-19 vaccination than their unemployed counterparts [8,17]. In our setup, multiple factors are possibly contributing to it including differences in knowledge, financial status and social communication, and higher frequency of females in the unemployed group. Our participants with lower income were more hesitant, this phenomenon was also seen in other parts of the world [9,24]. Again literacy may have played a role here. Another expected finding was those participants that have previously received any vaccination for other infectious diseases as an adult, were more willing for this vaccine as well. Our finding is consistent with published data [21,24]. In contrary to previous studies, we observed that respondents who had self or family exposure to COVID sickness have a strong intention for the vaccination, probably secondary to their fear and experience of the disease [9].

Finally, yet very importantly, 75% of our acceptant sample took the decision for vaccination by themselves after considering the importance and benefits of the vaccine. Although in a small percentage, the second most influential factor for acceptance was advice from a friend or family member, this was followed in third place by job requisites. Around the world, a persistent barrier of non-trust and fear of side effects have been strongly linked with COVID-19 vaccine hesitancy [6,10,21]. Similarly, the doubt about vaccines efficacy, its authenticity and fear of vaccine adverse effects were the most common stated reason for hesitancy in our study. Surprisingly, a significant number (37%) of participants still conveyed disbelief in the existence of at least of the reported severity of illness of COVID-19. 

Study limitation

Our survey had a limitation as these were the observation at a given point in time in the background of rapidly changing disease dynamics. Despite this limitation, our study reported some interesting findings which could be advantageous in setting targets and eliminating the barriers to COVID-19 vaccinations and will probably be helpful to similar vaccination programs.

## Conclusions

In the face of the devastating impact of COVID-19, the acceptance rate of the COVID vaccine is dismayingly low in our region, affected by the complex interaction of various socio-demographic and behavioral factors. A well-thought-out and targeted approach is crucial to mitigate the barriers to COVID-19 vaccination and allow a successful vaccination campaign to proceed in the country for the ultimate cessation of this pandemic and restoration of normal life.

## Appendices

PROFORMA

Participant Details

SNO:                _______________________              Date:   _______________________

Age (years):     ______________________               

Gender:                                                Male           Female

Marital Status:                                   Married      Unmarried  

Residence status:                                Rural          Urban

Ethnicity:                                             

Education:                                   

 No Formal Education (Never went to school)

 Primary (Class1-5)

 Secondary (Class 6-Matric)

 Higher (Intermediate to graduation)

Occupation:                 Medical      Non Medical    Unemployed

For Medical Professionals:

 Doctors      Nurses        Technicians       Porters            Janitors      Guards

 Administrators      Others      

Socioeconomic class:   

 Low (≤ 25000 per month)

 Middle (25000-50000 per month)

 High (≥ 50000 per month)

Previously (Other Than Covid-19) Vaccinated:        Yes              No

Had Covid-19                                                              Yes              No              When

Family Member affected with COVID-19:                 Yes              No              When

If COVID-19 Vaccine is freely provided by the Government, Will you go for COVID-19 vaccination?                                                   Yes              No              Not sure

If No/ Not sure? What is the reason for not getting vaccinated?

Unaware of COVID-19                                                        Religious Constrain  

Didn’t believe in COVID-19 existence or severity             Family Constraint

Don’t trust / threaten of vaccine                                         Medical reasons

Past Self COVID-19 Infections                  

COVID-19 Infected Family members with minor illness

Others (please specify; ______________________________________________)

If Yes? What is the reason of getting vaccinated?

Job requirement                                                                 Travelling requirement   

Self willingness                                                                   Family / friend advice     

 Past Self COVID-19 Infections                                           COVID-19 Infected Family                                                                                                             members       

Others (please specify; ______________________________________________)

Signature of Investigator: ______________             Patient Signature:      _________
